# Supplementary material for: Novel AIEgen-Functionalized Diselenide-Crosslinked Polymer Gels as Fluorescent Probes and Drug Release Carriers
Source: Polymers (Basel). 2020 Mar 3;12(3):551. doi: 10.3390/polym12030551 (PMC7182929; doi:10.3390/polym12030551)
Supplement: Supplementary file 1 [file polymers-12-00551-s001.pdf]

Novel AIEgen-functionalized Diselenide-Crosslinked Polymer Gels as Fluorescent Probes and Drug Release Carriers

Jie Zhao, Xiangqiang Pan *, Jian Zhu * and Xiulin Zhu

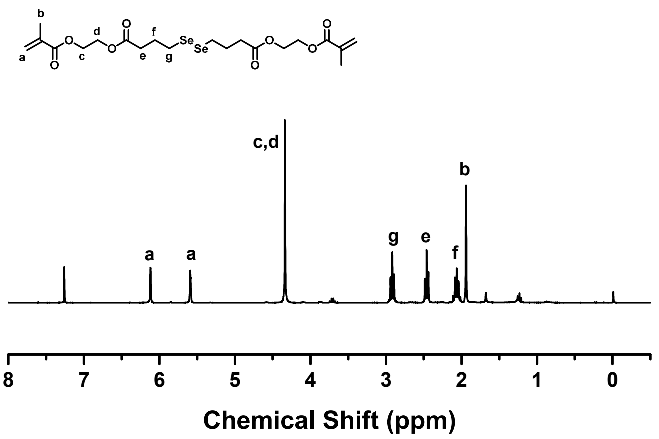

(a)

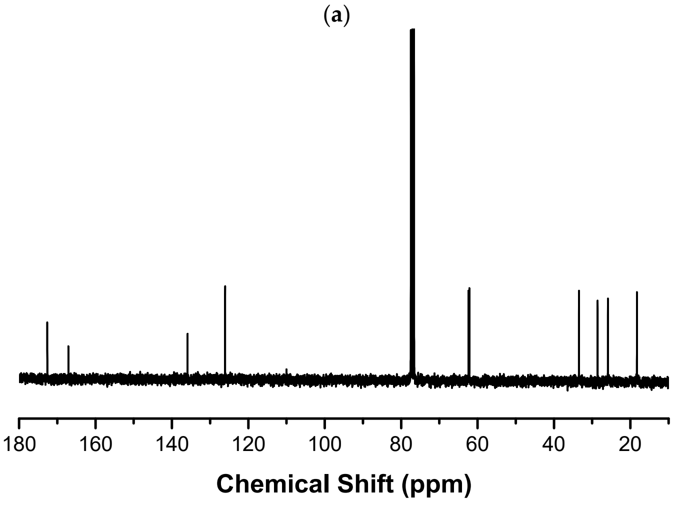

(b)

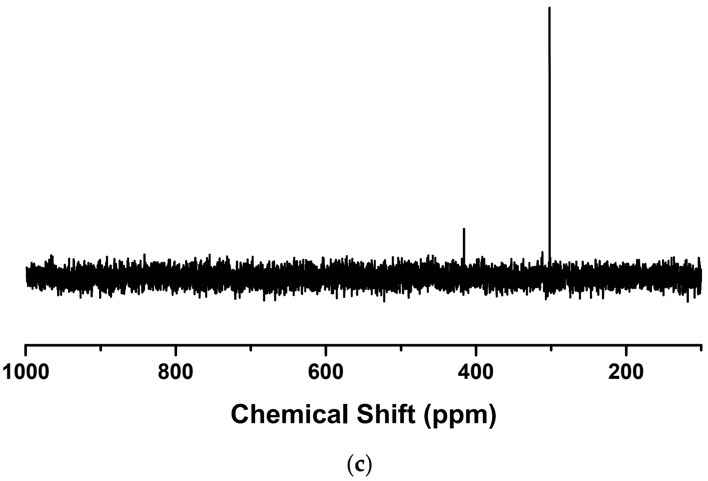

(c)

Figure S1. (**a**) ^{1}H NMR (**b**) ^{13}C NMR (**c**) ^{77}Se NMR spectrum of $(HEMA\text{-}Se)_2$ in $CDCl_3$.

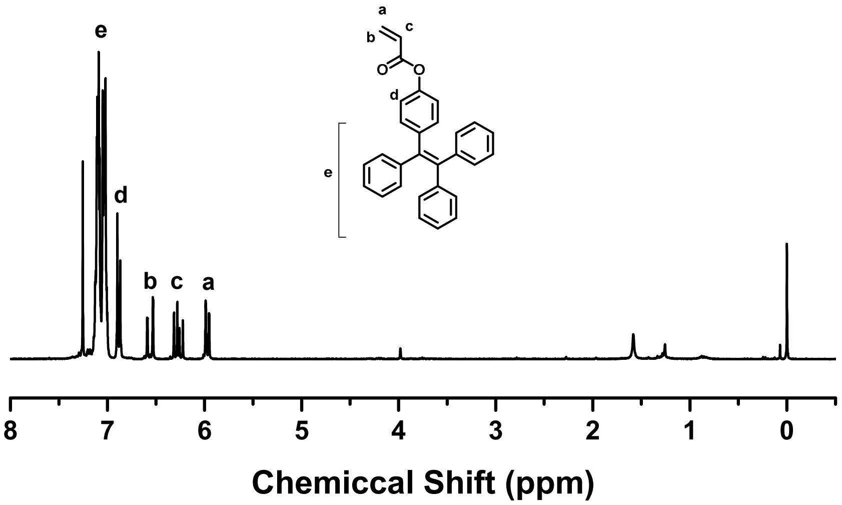

Figure S2. ^{1}H NMR spectrum of TPE-a in $CDCl_3$.

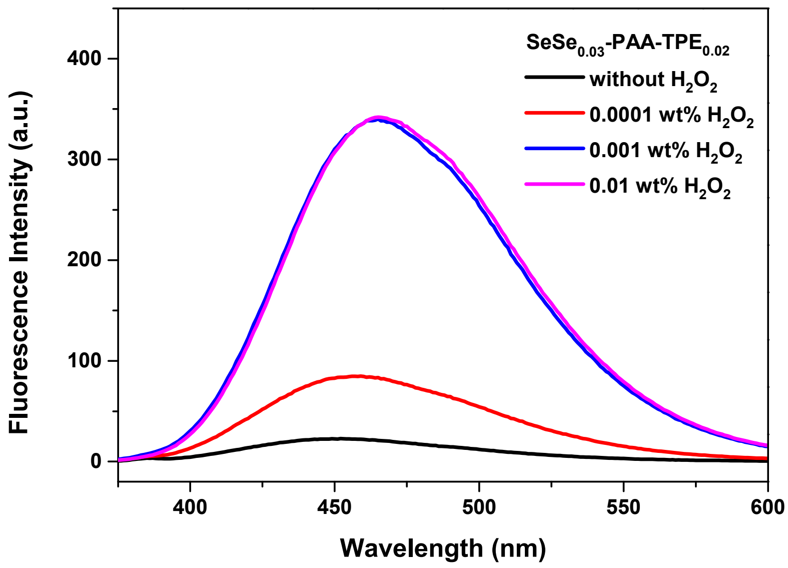

(a)

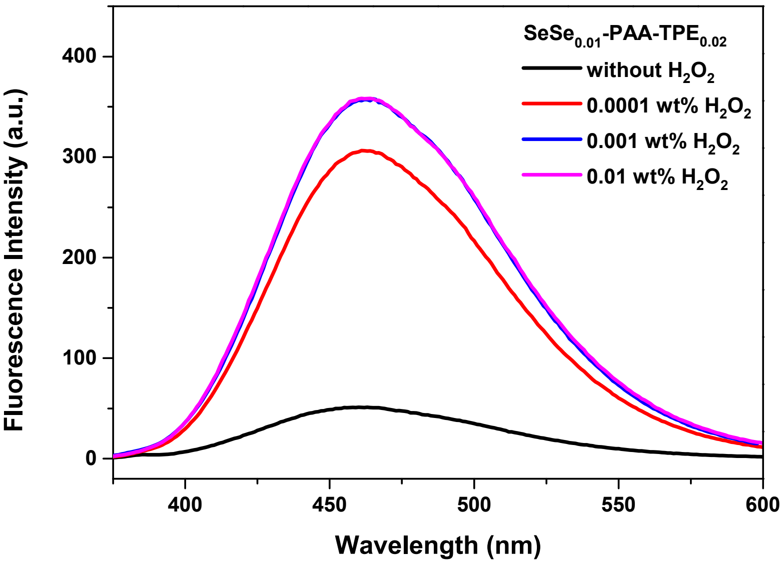

(**b**)

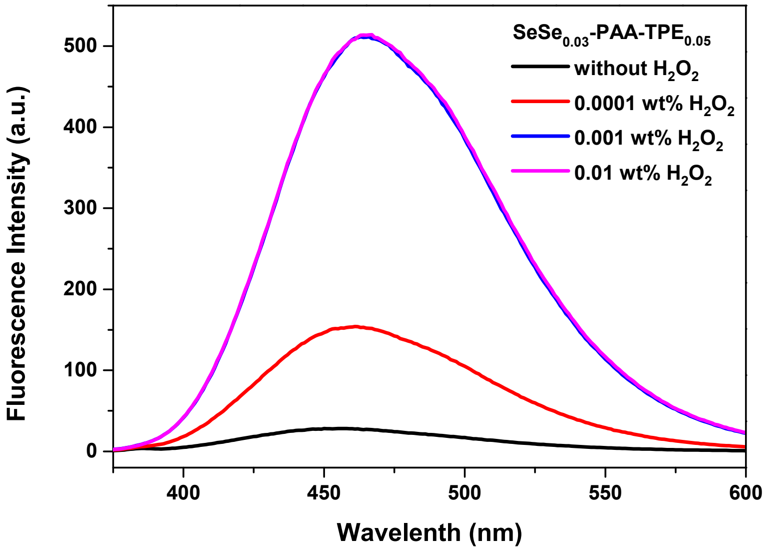

(**c**)

Figure S3. Fluorescence spectra (λ_{ex} = 340 nm) of the reaction solution that three polymer gels (**a**) $SeSe_{0.03}$-PAA-$TPE_{0.02}$; (**b**) $SeSe_{0.01}$-PAA-$TPE_{0.02}$; (**c**) $SeSe_{0.03}$-PAA-$TPE_{0.05}$ reacted with or without different H_2O_2 concentrations in PB solution (1 M, pH =7.4) respectively at room temperature.

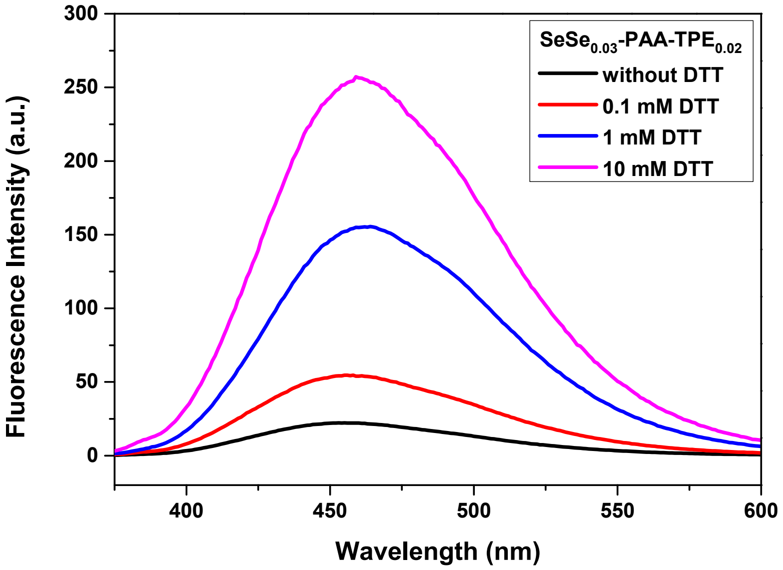

SeSe0.03-PAA-TPE0.02
without DTT
0.1 mM DTT
1 mM DTT
10 mM DTT
Fluorescence Intensity (a.u.)
Wavelength (nm)

(**a**)

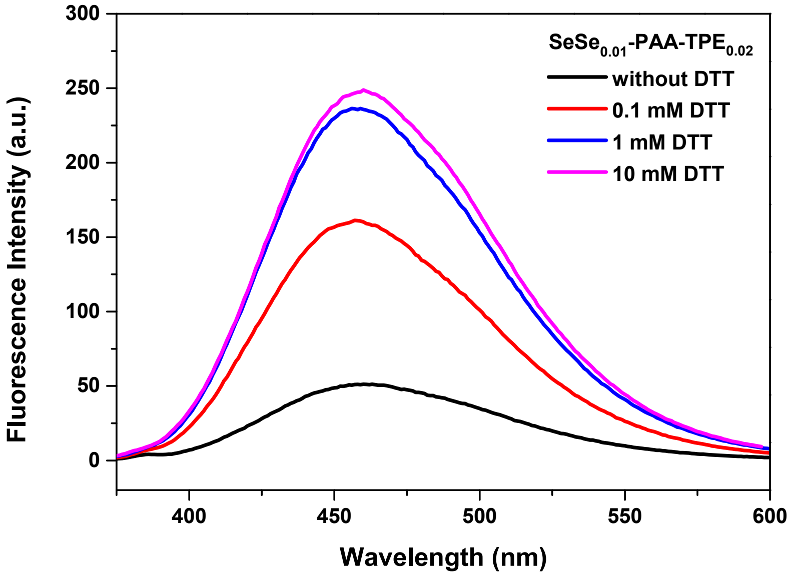

SeSe0.01-PAA-TPE0.02
without DTT
0.1 mM DTT
1 mM DTT
10 mM DTT
Fluorescence Intensity (a.u.)
Wavelength (nm)

(**b**)

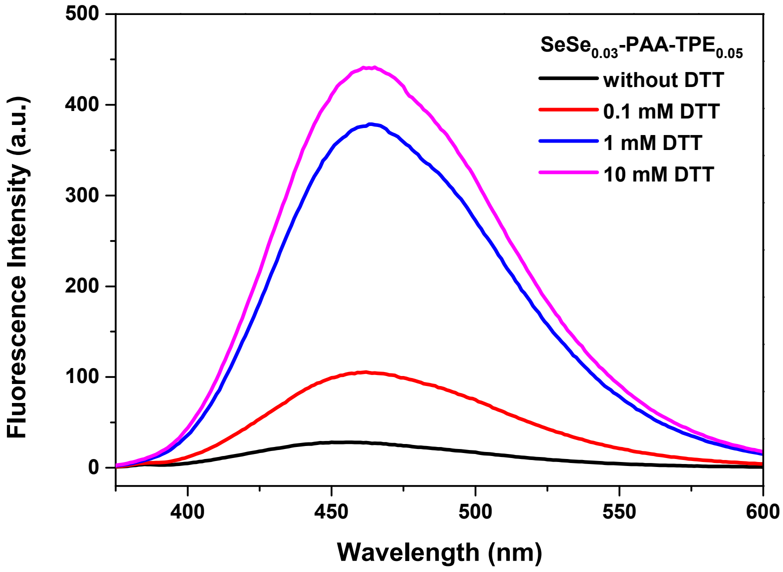

(**c**)

Figure S4. Fluorescence spectra (λ_{ex} = 340 nm) of the reaction solution that three polymer gels (**a**) $SeSe_{0.03}$-PAA-$TPE_{0.02}$; (**b**) $SeSe_{0.01}$-PAA-$TPE_{0.02}$; (**c**) $SeSe_{0.03}$-PAA-$TPE_{0.05}$ reacted with or without different DTT concentrations in PB solution (1 M, pH =7.4) respectively at room temperature.

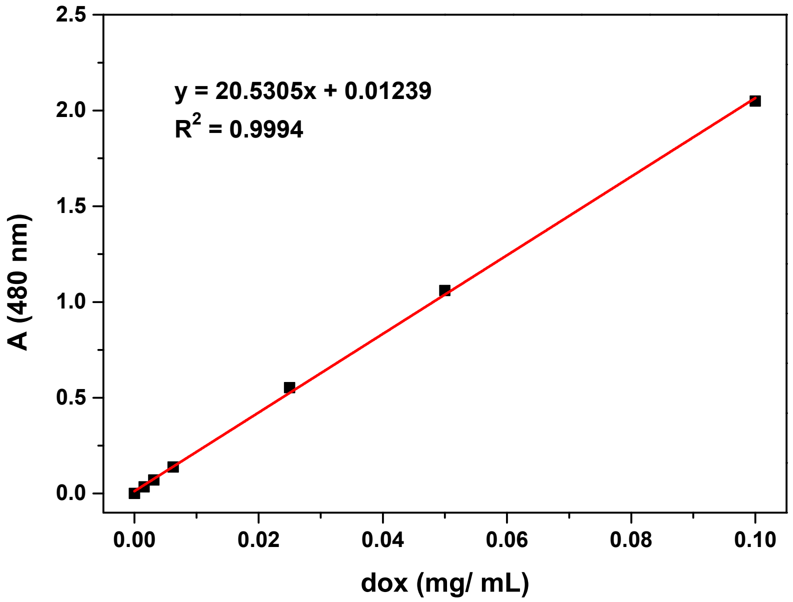

Figure S5. The calibration curve of different concentrations of DOX/ PB solution.
